# Use of Cardiac Procedures in People with Diabetes during the COVID Pandemic in Spain: Effects on the In-Hospital Mortality

**DOI:** 10.3390/ijerph20010844

**Published:** 2023-01-02

**Authors:** Ana Lopez-de-Andres, Rodrigo Jimenez-Garcia, David Carabantes-Alarcon, Valentín Hernández-Barrera, José M. de-Miguel-Yanes, Javier de-Miguel-Diez, Jose J. Zamorano-Leon, Jose L. del-Barrio, Natividad Cuadrado-Corrales

**Affiliations:** 1 Department of Public Health & Maternal and Child Health, Faculty of Medicine, Universidad Complutense de Madrid, 28040 Madrid, Spain; 2 Preventive Medicine and Public Health Teaching and Research Unit, Health Sciences Faculty, Universidad Rey Juan Carlos, Alcorcón, 28922 Madrid, Spain; 3 Internal Medicine Department, Hospital General Gregorio Marañón, Universidad Complutense de Madrid, Instituto de Investigación Sanitaria Gregorio Marañón (IiSGM), 28007 Madrid, Spain; 4 Respiratory Department, Hospital General Gregorio Marañón, Universidad Complutense de Madrid, Instituto de Investigación Sanitaria Gregorio Marañón (IiSGM), 28007 Madrid, Spain

**Keywords:** diabetes, cardiac procedures, hospitalization, COVID-19, in-hospital mortality

## Abstract

We aimed to assess the effect of the COVID-19 pandemic in Spain on people with diabetes undergoing cardiac procedures, such as coronary artery bypass graft (CABG), percutaneous coronary intervention (PCI), open surgical valve replacement (OSVR), and transcatheter valve implantation (TCVI). We compared the year 2019 with the year 2020. We conducted an observational study using data from the Spanish National Hospital Discharge Database from 1 January 2019 to 31 December 2020. In 2020, a total of 21,067 cardiac procedures were performed on people with diabetes compared with 24,675 in the previous year. The use of CABG, PCI, OSVR and TCVI decreased from 2019 to 2020 by 13.9%, 14.8%, 21.4% and 2.9%, respectively. In 2020, patients had a significantly higher mean Charlson Comorbidity Index than in 2019 for all the cardiac procedures analyzed. In-hospital mortality (IHM) was higher (*p* > 0.05) for all the procedures in the year 2020. Over the entire period, female sex was a significant risk factor for IHM among those who underwent CABG, PCI and OSVR (OR 1.94, 95%CI 1.41–2.66; OR 1.19, 95%CI 1.05–1.35; and OR 1.79, 95%CI 1.38–2.32, respectively). The sensitivity analysis including two more years, 2017 and 2018, confirmed that female patients and comorbidity were risk factors for IHM in patients with diabetes regardless of whether it was during the pandemic era or before. We conclude that the frequency of cardiac procedures among people with diabetes declined in 2020. IHM did not change significantly in the COVID-19 era.

## 1. Introduction

The COVID-19 pandemic has had a dramatic impact on healthcare systems, limiting access to services for patients with chronic conditions and affecting the care of patients with cardiovascular disease and the management of diabetes [1,2,3]. Most elective invasive cardiac procedures were cancelled or postponed, and several indications for non-elective surgical procedures were modified [4,5]. In a report for the year 2020, the Spanish Society of Cardiology found that several cardiovascular procedures had decreased markedly compared with the year 2019, in particular, percutaneous coronary interventions (PCI), which decreased by 9.4%, and transcatheter aortic valve replacements, which decreased by 0.9% [6].

Cardiovascular diseases are major causes of comorbidity and death among people with diabetes. In patients with cardiovascular diseases, those with concomitant diabetes more frequently have comorbidities and die [7]. For this reason, and because of the COVID-19 pandemic, postponing cardiovascular procedures may have had a specific impact on people with diabetes. Moreno et al. [8] concluded that among patients whose elective invasive cardiac procedure is cancelled or postponed, the clinical outcome is worse in those with diabetes, with higher all-cause mortality and cardiovascular mortality rates in the short term, irrespective of other clinical conditions.

Spain was one of the most affected countries during the first wave of COVID-19 in spring 2020 and globally in the year 2020 [9]. According to official data provided by the Instituto de Salud Carlos III, the number of confirmed infected persons that year was 1,907,853, and 193,597 (10.15%) of those were hospitalized [10]. The Spanish National Statistics Institute has published that in the year 2020 the total number of deaths with confirmed COVID-19 were 60,358, and this total is 14,481 more with suspected infections (for a total of 74,839) [11]. In response to the pandemic, Spain implemented some of the strictest lockdown measures in Europe. A nationwide state of emergency was declared on 14 March 2020 and ended on 21 June 2020. This period included strict home confinement, only allowing people to leave to obtain essential goods such as food or medicines or to go to health centres, and the government issued a halt to all non-essential activities. These measures were later eased, although mobility limitations were maintained throughout 2020, which affected accessibility to health care centres [12,13,14].

Reliable data obtained from national hospital discharge databases can be useful for identifying the effect of the COVID-19 pandemic on the use of cardiac procedures and hospital outcomes among people with diabetes.

This study used records of hospital admissions in Spain with the following objectives: (1) to assess changes over time (2019 vs. 2020) in the use of cardiac procedures among patients with diabetes; and (2) to analyse the effect of the COVID-19 pandemic on the sociodemographic and clinical characteristics and hospital outcomes of people with diabetes who underwent cardiac surgery in 2019 and 2020.

## 2. Materials and Methods

### 2.1. Study Design and Data Source

We conducted an observational study using data collected in the Spanish National Hospital Discharge Database (RAE-CMBD, *Registro de Actividad de Atención Especializada. Conjunto Mínimo Básico de Datos*, Registry of Specialized Health Care Activities. Minimum Basic Data Set). The RAE-CMBD was implemented in Spain in 1992. It was initially mandatory for public hospitals, but since 2016 private hospitals have also been obligated to report data. The Spanish Ministry of Health (SMH) is responsible for data collection, data depuration and quality assurance. It is estimated that over 95% of all hospital admissions are included in the database. Details on the RAE-CMBD methodology have been previously described [15,16,17].

The information collected by the RAE-CMBD comprises age, sex, place of residence, dates of admission and discharge, discharge destination (home, other social or medical institution, voluntary discharge, or deceased), primary diagnosis, secondary diagnosis (up to 19) and therapeutic and diagnostic procedures during hospitalization (up to 20). Coding in the RAE-CMBD is based on the International Classification of Disease 10th Revision (ICD-10). The RAE-CMBD is fulfilled by professional coders working at the hospital where the patient is admitted. These coders decide which ICD-10 codes are introduced in the registry using the diagnosis and procedures that the physician responsible for the patient has included in the discharge report and the information available in the hospital database. Coders receive regular training and updates from the SMH.

We analyzed data from the year 2019 (1 January 2019 to 31 December 2019) and from all of 2020 (1 January 2020 to 31 December 2020).

### 2.2. Study Population and Participants

The study population comprised patients aged 18 years or over admitted to a hospital with a diagnosis code for diabetes in their discharge records. The ICD-10 codes used to identify the study population are shown in Table S1.

Based on the ICD-10 procedure codes detailed in Table S1, discharges were grouped by cardiac procedure status as follows: (i) coronary artery bypass graft (CABG); (ii) PCI; (iii) open surgical aortic, mitral or tricuspid or pulmonary valve replacement (OSVR); and (iv) transcatheter aortic or mitral or tricuspid or pulmonary valve implantation (TCVI).

The exclusion criteria were as follows: (i) missing data for age, sex, place of residence, dates of admission or discharge and discharge destination; (ii) both codes of CABG and PCI in any therapeutic procedures field; (iii) a code for other cardiac procedures such as replacement of the ascending aorta, closure of ventricular and atrial septal defects, ablation and other rare procedures in any therapeutic procedures field; and (iv) more than one admission for the same patient during the study period.

### 2.3. Study Variables

The main outcome variables of this investigation were the difference in the use of cardiac procedures and in-hospital mortality (IHM) among patients with diabetes between 2019 and 2020. The IHM is defined as the percentage of hospitalized patients who died during admission.

Secondary outcome variables were admission (yes/no) to the intensive care unit (ICU), length of ICU stay, and total length of hospital stay (LOHS).

To assess global comorbidity, the mean number of conditions included in the Charlson Comorbidity Index (CCI) was calculated using the algorithms previously proposed by Sundararajan et al. [18]. The specific conditions analyzed were peripheral vascular disease, ischaemic heart disease, valvular heart disease, atrial fibrillation, heart failure, chronic kidney disease, and COVID-19. Procedures included the use of non-invasive and invasive mechanical ventilation and dialysis. Table S1 shows the clinical conditions and therapeutic procedures analyzed and the ICD-10 codes used to identify them.

### 2.4. Statistical Methods

The descriptive analysis was performed with the calculation of means with standard deviation for quantitative variables and with absolute and relative frequencies, expressed as percentages, for qualitative variables.

The change from the year 2019 to the year 2020 in the use of the four procedures was expressed as a proportion, obtained by subtracting the number of procedures performed in 2020 from those conducted in 2019, divided by the first, and multiplied by one hundred [(no. in 2019–no. in 2020/no. in 2019) × 100].

When comparing the demographic and clinical conditions between 2019 and 2020, we used the t-test or the Mann-Whitney test for means and the chi square test for proportions.

To control the possible effect of changes in the distribution by age, sex, and comorbidity on the IHM from 2019 to 2020, we applied multivariable logistic regression models, generating one model for each procedure. The models were constructed using the recommendations of Hosmer et al. [19]. Two-way interactions were examined.

### 2.5. Sensitivity Analysis

To control possible fluctuations in the number and outcomes of cardiac procedures over the years we have also analyzed the results for years 2017 and 2018, using identical methods to those described for years 2019 and 2020. We did not include year 2016 because that year was the first one the RAE-CMBD used the ICD10, and according to the SMH there was a significant under-notification. To assess the temporal trend on study variables we used a linear regression t-test for means and the Cochran-Armitage test for proportions. The logistic regression model for IHM was repeated including the same variables previously mentioned and the years 2017 to 2020.

Data were analysed using Stata 14. A *p* < 0.05 (two-tailed) is considered statistically significant.

### 2.6. Ethical Aspects

The RAE-CMBD database can be requested from the SMH [20], which evaluates the proposal. If this is considered adequate from the scientific and ethical point of view, anonymised records are sent. Therefore, the study protocol was not evaluated by an ethics committee, and, as this is an administrative database, informed consent was not needed from the participants.

## 3. Results

According to the RAE-CMBD, in the first year of the COVID-19 pandemic and the lockdown period in Spain, there were 21,067 cardiac procedures in patients with diabetes aged ≥18 years. Men accounted for 72.6% (15,299) and women 27.4% (5768). The total number of procedures the previous year was 24,675; a 14.6% reduction.

The use of cardiac procedures among people with diabetes by months for the years 2019 and 2020 is shown in Figure S1. Compared with the year 2019, the frequency of the cardiac procedures studied decreased markedly between the date of lockdown (14 March 2020) and the end of lockdown in Spain (2 May 2020). Furthermore, the frequency of CABG, PCI and OSVR decreased in September, October, November and December of 2020, corresponding with the second wave of COVID-19 in Spain [10].

### 3.1. Clinical Characteristics and Hospital Outcomes according to Cardiac Procedures

Table 1 shows the distribution of people with diabetes who underwent CABG according to sociodemographic characteristics, procedures and in-hospital outcomes. In 2020, the number of patients with diabetes in Spain who underwent CABG fell by 13.9% (2780 in 2019 to 2394 in 2020). In 2020, patients had a higher mean CCI than in 2019 (2.6 vs. 2.5, *p* = 0.003), as well as a significantly higher prevalence of heart failure (20.3% in 2020 vs. 18% in 2019; *p* = 0.035). The percentage of hospitalized patients with diabetes admitted to the ICU after CABG was 84.0% in 2020 and 79% in 2019 (*p* < 0.001). IHM was slightly, but not significantly, higher in 2020 (4.1% vs. 3.8%; *p* = 0.553).

The number of PCI conducted among people with diabetes was 15,560 in 2020 and 18,255 in 2019, which amounts to a decrease of 14.8% in 2020 (Table 2). The mean CCI was higher in the year 2020 than in 2019 (2.9 vs. 2.8; *p* < 0.001). Specifically, the prevalence of valvular heart disease, heart failure, and chronic kidney disease was higher in 2020. LOHS was longer (6.4 ± 8 days vs. 6.8 ± 9.4 days) in 2020 than in 2019 (*p* = 0.001). As for CABG, the differences in IHM did not reach statistical significance (3.9% in 2020 vs. 3.5% in 2019; *p* = 0.88).

As seen in Table 3, the number of hospitalizations in people with diabetes who underwent OSVR fell by 21.4% between 2019 and 2020 (2280 in 2019 vs. 1792 in 2020). The year 2020 differed from 2019 in that people with diabetes had a higher mean CCI (2.4 vs. 2.2; *p* < 0.001) and more frequently had ischaemic heart disease (42.5% vs. 38.3%; *p* = 0.006), atrial fibrillation (42.9% vs. 39.7%; *p* = 0.041), heart failure (24.6% vs. 21.5%; *p* = 0.019) and chronic kidney disease (17.1% vs. 14%; *p* = 0.006). The proportion of hospitalized patients admitted to the ICU was higher in 2020 than in 2019 (83.2% vs. 77.3%; *p* < 0.001).

The IHM was 6.1% in 2019 and 6.8% in 2020 (*p* = 0.429).

Between 2019 and 2020, the number of hospitalizations in patients with diabetes who underwent TCVI fell by 2.9% (1360 in 2019 vs. 1321 in 2020). As can been seen in Table 4, higher values were recorded in 2020 for mean CCI (2.8 vs. 2.6; *p* < 0.001) and the prevalence of ischaemic heart disease (40.58% vs. 35.9%; *p* = 0.012). Similar figures were found for IHM (2.9% in 2019 vs. 3.2% in 2020; *p* = 0.720).

### 3.2. Multivariable Analysis of IHM in People with Diabetes Hospitalized in Spain, 2020, According to Cardiac Procedures

As seen in Table S2, the results of the multivariable logistic regression analysis revealed no significant association between year of hospitalization (2019 vs. 2020) and IHM in people with diabetes who underwent any of the four cardiac procedures investigated.

Female sex was a significant risk factor for IHM among those who underwent CABG, PCI and OSVR (adjusted OR 1.94, 95%CI 1.41–2.66; adjusted OR 1.19, 95%CI 1.05–1.35; and adjusted 1.79, 95%CI 1.38–2.32, respectively). For all of the procedures, the risk of dying in hospital increased with age. The presence of comorbidity, measured with the CCI, increased the risk of death during hospitalization in patients who underwent CABG (adjusted OR 1.45, 95%CI 1.35–1.55), PCI (adjusted OR 1.32, 95%CI 1.28–1.36), OSVR (adjusted OR 1.36, 95%CI 1.27–1.47) and TCVI (adjusted OR 1.26, 95%CI 1.12–1.42).

### 3.3. Sensitivity Analysis

The number of the four cardiac procedures in 2017, 2018 and 2019 can be seen in Table S3. For CABG, the number of procedures was very similar in these years, with figures of around 2780. Furthermore, no significant changes were found for the distribution according to age. However, the CCI showed a small, but significant, increase. The number of PCI has increased from 16,437 in 2017, to 17,422 in 2018 and 18,255 in 2019. The mean age and CCI also rose significantly. Regarding OSVR, the number of interventions remained stable from 2017 to 2019 without any change in the population characteristics. As reported for PCI, the use of TCVI has been constantly increasing from 2017 to 2019 (791 vs. 1360) with sex distribution, age and CCI showing no significant variation in overtime.

The IHM for the four cardiac procedures in the three-years prior to the COVID-19 pandemic year can be seen in Table S4. For CABG and TCVI, no significant changes were observed over time. PCI mortality rose slightly from 2017 to 2019 for both sexes and for women and men separately. In addition, a small but significant increment for IHM after OSVR was seen in both women and men.

When the IHM according to sex was analyzed in the years 2017 to 2020, it was found that women had significantly higher percentages for PCI in all years, three years for CABG and two years for OSVR. There were no differences according to sex in the IHM after TCVI in any year studied.

The results of the multivariable model introducing the people with diabetes who underwent these procedures in the years 2017 and 2020 can be seen in Table S5. The results remain similar to those found when only the years 2019 and 2020 were compared, showing that being a woman (except for TCVI), of older age and with higher CCI were risk factors for IHM in all the surgical cardiac procedures analyzed. No significant changes in IHM overtime were found in any procedure.

## 4. Discussion

This nationwide registry and population-based observational study of changes in the activity of cardiac procedures during the COVID-19 pandemic revealed several key findings. First, during the lockdown imposed on 14 March 2020, there was an important decline in all procedures performed in patients with diabetes in Spain. In the second wave of COVID-19, between September and December 2020, the activity of CABG, PCI and OSVR also decreased. Second, during the COVID-19 pandemic year, patients with diabetes who underwent cardiac procedures had more comorbidities. Third, while IHM was higher in all cardiac procedures, the difference was not significant. Finally, besides the year analyzed, female sex was associated with a 94%, 79% and 19% higher risk of dying in hospital than men following CABG, OSVR and PCI, respectively.

Several studies have already reported changes in cardiac surgical activity during the COVID-19 pandemic [4,21,22]. A retrospective cohort study of procedures recorded in the Spanish Infarct Code Registry revealed a drop of 27.6% in patients treated for STEMI between the pre-COVID and COVID eras [23]. Indeed, data from several Spanish centres suggest an overall decrease of 48% in PCI and a 40% decrease in PCI in patients with ST-Elevation Myocardial Infarction (STEMI) [24]. In the UK, Day et al. [1] found a dramatic decline in the number of acute referrals and associated procedures at the start of the pandemic, an observation that has been corroborated elsewhere. Other authors have hypothesized that many STEMI patients avoided or delayed initiation of care during the COVID-19 era [25].

Among people with diabetes, our results are in line with those reported in the literature, namely, a consistent decrease in the number of cardiac procedures performed during the COVID-19 pandemic. Furthermore, data from the International Study on Acute Coronary Syndromes-ST Elevation Myocardial Infarction (ISACS-STEMI) show that during the COVID-19 pandemic, significantly fewer PCI were performed than in 2019; this finding was similar for patients with and without diabetes (IRR 0.79, 95%CI 0.73–0.85 and IRR 0.81, 95%CI 0.78–0.85) [26].

As can be seen in Figure S1, in Spain the most remarkable reduction in the number of procedures was observed in the lockdown months. However, even if hospitals tried to catch up in the summer months, the appearance of the second wave in Spain, from the end of June to the first week of December 2020, also resulted in a reduction of most procedures in the last six months of that year.

Our study provides information about the risk profile of patients with diabetes who underwent cardiac surgery during the COVID-19 pandemic. Patients with diabetes had a worse clinical profile, including a higher prevalence of some comorbidities such as heart failure in the case of patients undergoing CABG or PCI, and ischaemic heart disease in patients who underwent OSVR and TCVI, as reported elsewhere [8].

For the most common cardiac procedures, Day et al. [1] found that 30-day all-cause mortality (HR = 0.76; 95%CI 0.27–2.20) in the general population was similar in the COVID era to values recorded during the previous five years. We agree with Day et al., finding that, in all cardiac procedures done on patients with diabetes admitted in 2020 and 2019, no significant differences in the IHM exist before and after adjustment for possible confounders [1]. Our data is in line with the results of the ISACS-STEMI COVID-19 study, which found a significantly higher mortality rate in 2020 than in 2019 (68 deaths [11.4%] vs. 60 deaths [7.9%], OR [95% CI]  =  1.5 [1.04–2.16], *p*  =  0.03), although this association was not significant after correction for all potential confounding factors (OR 1.36, 95%CI 0.93–1.99). The authors concluded that there may be a delay from symptom onset to first medical contact, and that this may be due to factors associated with the patient or with the emergency system [26,27,28]. Furthermore, the higher IHM (prior to adjusting for the covariates) could also reflect the fact that due to the limited healthcare services available during the pandemic, only the most severe cases were admitted to the hospital for cardiac surgery.

We found that female sex and the presence of comorbidity were risk factors for mortality in patients with diabetes who underwent cardiac procedures during the COVID-19 era. We also found that older age was a risk factor for IHM in patients undergoing PCI. These predictors of worse outcomes after a cardiac surgical procedure have been reported elsewhere [29,30,31]. The sensitivity analysis including two more years (2017 and 2018) showed that female patients and the presence of comorbidity were risk factors for mortality in patients with diabetes regardless of whether it was during the pandemic era or not.

Several practical implications can be derived from our investigation. It is necessary that health services prepare or update protocols to guarantee the prioritization of cases to be operated and therapeutic alternatives to surgery for patients with lower risk. Is also important to optimize the health organization so patients can be discharged as soon as possible to reduce their risk of being infected and to have free beds for new patients. Furthermore, improvements are required in terms of coordination at the national or regional levels so that all health resources are efficiently used, considering the creation of centres dedicated exclusively to the treatment of positive or disease-free patients [5,8,32,33]. In our country, other authors have proposed new programs, including surgery in the afternoon and increasing the surgical activity in the summer months [5,8,32,33]. In primary care centres, better funding and more human resources are necessary to increase the capacity of primary care networks, and further efforts are needed to maximize professional competencies, make meaningful use of digital solutions, and to implement more efficient administrative processes [34].

Future research should include the analysis of the entire COVID-19 pandemic, not only the year 2020, and studies should be done with more extensive and precise clinical and socio-demographic data to confirm our results. Furthermore, the physiological consequences of the COVID-19 pandemic and their effect on lifestyles, self-care and use of health services should be investigated using quantitative and qualitative methods. Finally, investigations to identify pitfalls and to improve the surveillance and organizational systems, including digital health solutions, are necessary.

Even if previous studies have reported the impact of COVID-19 on patients’ access to healthcare resources, we think that the following factors make our investigation novel and important. First, to this point in time, few studies have focused specifically on people with diabetes. This is relevant because this chronic disease is associated not only with a higher incidence of heart conditions but also with worse outcomes after surgical heart procedures. Second, we have used a database that provides data from almost all of the hospitalizations in an entire country of 47 million inhabitants, so the external validity of the results is very high, and the selection bias that can affect studies including registers from a limited number of hospitals is avoided. Third, Spain has been one of the European countries more strongly hit by the first waves of COVID-19, and the results of our investigation can help other countries to prepare for future pandemics. Fourth, one strength of our investigation is that we analysed global comorbidity, with the CCI, specific clinical baseline conditions and in-hospital procedures that have been previously associated to the outcomes of heart procedures. Fifth, the RAE-CMBD has been conducted in Spain since 1992 with a standardized methodology and the quality of the data is ensured through annual assessments. Finally, the validity of the coding applied for diabetes and cardiac procedures in the RAE-CMBD has been reported previously [35,36,37,38].

Nevertheless, our study is subject to a series of limitations. First, our data was obtained from an administrative database containing information recorded by physicians in the discharge report; therefore, data on clinical characteristics, glycemic control, medical treatments, and duration of diabetes are not recorded. Second, as the study was restricted to relatively low-risk procedures, caution is needed when extrapolating our conclusions to higher-risk cardiac procedures, or indeed to other surgical disciplines [1]. Third, the low risk of the procedures studied means that the comparison in the IHM was conducted with small sample sizes and that mortality was not assessed once the patient left the hospital. Fourth, the lower number of procedures in summer has been previously described in Spain, and the main explanation for this is that hospital beds are closed for the summer vacations of health personnel. Fifth, the RAE-CMBD does not include information on the reason the surgery was not conducted, (such as patient refusal or postponement or cancellation by the health services). However, studies conducted in Spain have concluded that most surgeries were postponed or cancelled by the health authorities due to the overloading of the hospitals and particularly the intensive care units with COVID-19 patients [5,8,32,33].

Sixth, the adequate control of confounding poses a challenge in studies that use health care databases, since these were not designed for undertaking epidemiologic studies. Unfortunately, the RAE-CMBD does not collect information on sociodemographic variables such as marital status, household income, occupation or geographic location of the patients, which renders it impossible to adjust for such factors to control for confounding. The reason for this is lack of information is to protect the privacy of individual patients. Sociodemographic variables may have affected our results because previous investigations in Spain have reported that income, occupation and living alone or in deprived areas are factors associated with a higher probability of COVID-19 infection, higher psychological vulnerability, and poorer access to the healthcare system [39,40,41]. Future investigations should consider the effect of these variables, including them in the statistical models.

Besides the aforementioned limitations, the results of the sensitivity analysis showed that the results are reliable and remain similar when more years prior to 2019 are analyzed. Furthermore, for PCI and TCVI, the time trend from 2017 to 2019 suggests an important increment over time. As such, the effect of the COVID-19 pandemic on the use of these procedures is possibly more significant than reported.

## 5. Conclusions

In 2020, we recorded a decline in the number of cardiac procedures performed, although we observed that IHM was similar both before and during the COVID-19 pandemic, even if patients had more comorbid conditions.

## Figures and Tables

**Table 1:** Sociodemographic and clinical characteristics and in-hospital outcomes of patients with diabetes who underwent a coronary artery bypass graft in Spain in the years 2019 and 2020. Analysis of the Spanish National Hospital Discharge Database.

	2019	2020	*p*-Value	Difference * (2019 to 2020) %
N (%)	2780	2394		−13.9
Women n (%)	534 (19.2)	448 (18.7)	0.651	−16.1
Men n (%)	2246 (80.8)	1946 (81.2)		−13.4
Age. mean (SD)	67.74 (8.5)	67.44 (8.8)	0.214	−0.4
<50 years. n (%)	70 (2.5)	88 (3.7)	0.080	+20.5
50–64 years. n (%)	876 (31.5)	731 (30.5)		−16.6
65–79 years. n (%)	1664 (59.9)	1442 (60.2)		−13.3
≥80 years. n (%)	170 (6.1)	133 (5.6)		−21.8
CCI index. mean (SD)	2.49 (1.5)	2.62 (1.6)	0.003	+5.2
Type 2 diabetes n (%)	2720 (97.8)	2326 (97.1)	0.224	−14.5
Type 1 diabetes n (%)	60 (2.2)	68 (2.8)		+11.8
Peripheral vascular disease n (%)	341 (12.3)	307 (12.8)	0.546	−10
Valvular heart disease n (%)	887 (31.9)	781 (32.6)	0.582	−12%
Atrial fibrillation n (%)	581 (20.9)	540 (22.6)	0.149	−7.1
Heart failure n (%)	500 (18)	486 (20.3)	0.035	−2.8
Chronic kidney disease n (%)	426 (15.3)	378 (15.8)	0.645	−11.3
COVID-19 n (%)	NA	12 (0.5)	NA	NA
Non-invasive mechanical ventilation. n (%)	74 (2.7)	78 (3.3)	0.205	+5.1
Invasive mechanical ventilation. n (%)	391 (14.1)	334 (14)	0.907	−14.6
Dialysis. n (%)	108 (3.9)	88 (3.7)	0.695	−18.5
Admission to ICU. n (%)	2196 (79)	2012 (84.0)	<0.001	−8.4
LOHS. mean (SD)	17.11 (15.5)	16.57 (14.2)	0.284	−3.1
IHM. n (%)	106 (3.8)	99 (4.1)	0.553	−6.6

*p*-value comparing the proportions or means for years 2019 vs. 2020. * Difference obtained resting the higher absolute number of subjects for year 2019 or 2020 to the lower divided by the higher absolute number. Shown using 2019 as the reference. T2DM: type 2 diabetes mellitus; T1DM: type 1 diabetes mellitus CCI: Charlson comorbidity index; ICU Intensive care unit; SD Standard deviation; LOHS: length of hospital stays; IHM: in-hospital mortality. NA: Not applicable.

**Table 2:** Sociodemographic and clinical characteristics and in-hospital outcomes of patients with diabetes who underwent a percutaneous coronary intervention in Spain in 2019 and 2020. Analysis of the Spanish National Hospital Discharge Database.

	2019	2020	*p*-Value	Difference * (2019 to 2020) %
N (%)	18,255	15,560		−14.8
Women n (%)	4834 (26.5)	4029 (25.9)	0.221	−16.7
Men n (%)	13,421 (73.5)	11,531 (74.1)		−14.1
Age. mean (SD)	69.26 (10.9)	69.11 (11.0)	0.209	−0.211
<50 years. n (%)	777 (4.3)	656 (4.2)	0.690	−15.6
50–64 years. n (%)	5236 (28.7)	4554 (29.3)		−13.0
65–79 years. n (%)	8739 (47.9)	7372 (47.4)		−15.6
≥80 years. n (%)	3503 (19.2)	2978 (19.1)		−15
CCI index. mean (SD)	2.75 (1.6)	2.87 (1.6)	<0.001	+4.2
Type 2 diabetes n (%)	17,947 (98.2)	15,293 (98.1)	0.958	−14.8
Type 1 diabetes n (%)	308 (1.7)	268 (1.7)		−13
Peripheral vascular disease n (%)	1419 (7.8)	1294 (8.3)	0.67	−8.8
Valvular heart disease n (%)	2932 (16.1)	2718 (17.5)	0.001	−7.3
Atrial fibrillation n (%)	2453 (13.4)	2142 (13.8)	0.379	−12.7
Heart failure n (%)	3839 (21.0)	3527 (22.7)	<0.001	−8.1
Chronic kidney disease n (%)	3108 (17.0)	2837 (18.2)	0.004	−8.7
COVID-19 n (%)	NA	136 (0.9)	NA	NA
Non-invasive mechanical ventilation. n (%)	366 (2.0)	347 (2.23	0.151	−5.2
Invasive mechanical ventilation. n (%)	512 (2.8)	444 (2.9)	0.787	−13.3
Dialysis. n (%)	347 (1.9)	326 (2.1)	0.202	−6.1
Admission to ICU. n (%)	6266 (34.3)	5365 (34.5)	0.765	−14.4
LOHS. mean (SD)	6.78 (9.4)	6.42 (8)	0.001	−5.341
IHM. n (%)	639 (3.5)	599 (3.9)	0.88	−6.3

*p*-value comparing the proportions or means for years 2019 vs. 2020. * Difference obtained resting the higher absolute number of subjects for 2019 or 2020 to the lower divided by the higher absolute number. Shown using 2019 as the reference. T2DM: type 2 diabetes mellitus; T1DM: type 1 diabetes mellitus CCI: Charlson comorbidity index; ICU Intensive care unit; SD Standard deviation; LOHS: length of hospital stays; IHM: in-hospital mortality. NA: Not applicable.

**Table 3:** Sociodemographic and clinical characteristics and in-hospital outcomes of patients with diabetes who underwent an open surgical heart valve replacement in Spain in the years 2019 and 2020. Analysis of the Spanish National Hospital Discharge Database.

	2019	2020	*p*-Value	Difference * (2019 to 2020) %
N (%)	2280	1792		−21.4
Women n (%)	859 (37.7)	655 (36.6)	0.461	−23.8
Men n (%)	1421 (62.3)	1137 (63.5)		−20
Age. mean (SD)	70.44 (7.9)	70.60 (7.8)	0.519	+0.2
<50 years. n (%)	36 (1.6)	22 (1.2)	0.775	−38.9
50–64 years. n (%)	446 (19.6)	343 (19.1)		−23.1
65–79 years. n (%)	1577 (69.2)	1256 (70.1)		−20.4
≥80 years. n (%)	221 (9.7)	171 (9.5)		−22.7
CCI index. mean (SD)	2.21 (1.4)	2.37 (1.5)	<0.001	+6.7
Type 2 diabetes n (%)	2255 (98.9)	1779 (99.2)	0.467	−21.1
Type 1 diabetes n (%)	25 (1.1%)	13 (0.7)		−48.0
Peripheral vascular disease n (%)	253 (11.1)	208 (11.6)	0.610	−17.8
Ischemic heart disease n (%)	872 (38.3)	761 (42.5)	0.006	−12.7
Atrial fibrillation n (%)	906 (39.7)	769 (42.9)	0.041	−15.1
Heart failure n (%)	490 (21.5)	441 (24.6)	0.019	−10.0
Chronic kidney disease n (%)	319 (14)	307 (17.1)	0.006	−3.8
COVID-19 n (%)	NA	9 (0.5)	NA	NA
Non-invasive mechanical ventilation. n (%)	69 (3.0)	61 (3.4)	0.496	−11.6
Invasive mechanical ventilation. n (%)	428 (18.8)	335 (18.7)	0.950	−21.7
Dialysis. n (%)	96 (4.2)	93 (5.2)	0.140	−3.1
Admission to ICU. n (%)	1762 (77.3)	1491 (83.2)	<0.001	−15.4
LOHS. mean (SD)	17.50 (17.7)	16.88 (15.1)	0.278	−3.5
IHM. n (%)	140 (6.1)	121 (6.8)	0.429	−13.6

*p*-value comparing the proportions or means for years 2019 vs. 2020. * Difference obtained resting the higher absolute number of subjects for 2019 or 2020 to the lower divided by the higher absolute number. Shown using 2019 as the reference. T2DM: type 2 diabetes mellitus; T1DM: type 1 diabetes mellitus CCI: Charlson comorbidity index; ICU Intensive care unit; SD Standard deviation; LOHS: length of hospital stays; IHM: in-hospital mortality. NA: not applicable.

**Table 4:** Sociodemographic and clinical characteristics and in-hospital outcomes of patients with diabetes who underwent a trans-catheter valve implantation in Spain in the years 2019 and 2020. Analysis of the Spanish National Hospital Discharge Database.

	2019	2020	*p*-Value	Difference * (2019 to 2020) %
N (%)	1360	1321		−2.9
Women n (%)	644	636	0.681	−1.2
Men n (%)	716	685		−2.9
Age. mean (SD)	79.64 (6.8)	79.45 (6.75)	0.437	−0.2
<50 years. n (%)	3 (0.2)	5 (0.4)	0.760	+40
50–64 years. n (%)	33 (2.4)	34 (2.6)		+2.9
65–79 years. n (%)	546 (40.2)	547 (41.4)		+0.2
≥80 years. n (%)	778 (57.2)	735 (55.6)		−5.5
CCI index. mean (SD)	2.62 (1.6)	2.84 (1.7)	<0.001	+7.8
Type 2 diabetes n (%)	1355 (99.4)	1313 (99.3)	0.422	−3.1
Type 1 diabetes n (%)	5 (0.4)	8 (0.6)		+37.5
Peripheral vascular disease n (%)	141 (10.4)	160 (12.1)	0.153	+11.9
Ischemic heart disease n (%)	488 (35.9)	536 (40.6)	0.012	+9
Atrial fibrillation n (%)	485 (35.7)	483 (6.6)	0.627	−0.4
Heart failure n (%)	398 (29.3)	412 (31.2)	0.278	+3.4
Chronic kidney disease n (%)	346 (25.4)	363 (27.5)	0.232	+4.7
COVID-19 n (%)	NA	7 (0.5)	NA	NA
Non-invasive mechanical ventilation. n (%)	21 (1.5)	29 (2.2)	0.213	+27.6
Invasive mechanical ventilation. n (%)	33 (2.4)	41 (3.1)	0.285	+19.5
Dialysis. n (%)	26 (1.9)	31 (2.4)	0.435	+16.1
Admission to ICU. n (%)	636 (46.8)	657 (49.7)	0.124	+3.2
LOHS. mean (SD)	11.13 (11.2)	10.66 (10.2)	0.365	−4.2
IHM. n (%)	40 (2.9)	42 (3.2)	0.720	−4.8

*p*-value comparing the proportions or means for years 2019 vs. 2020. * Difference obtained resting the higher absolute number of subjects for 2019 or 2020 to the lower divided by the higher absolute number. Shown using 2019 as the reference. T2DM: type 2 diabetes mellitus; T1DM: type 1 diabetes mellitus CCI: Charlson comorbidity index; ICU Intensive care unit; SD Standard deviation; LOHS: length of hospital stays; IHM: in-hospital mortality. NA: not applicable.

## Data Availability

According to the contract signed with the Spanish Ministry of Health and Social Services, which provided access to the databases from the Spanish National Hospital Database (RAE-CMBD, *Registro de Actividad de Atención Especializada. Conjunto Mínimo Básico de Datos*, Registry of Specialized Health Care Activities. Minimum Basic Data Set), we cannot share the databases with any other investigator, and we have to destroy the databases once the investigation has concluded. Consequently, we cannot upload the databases to any public repository. However, any investigator can apply for access to the databases by filling out the questionnaire available at http://www.msssi.gob.es/estadEstudios/estadisticas/estadisticas/estMinisterio/SolicitudCMBDdocs/Formulario_Peticion_Datos_CMBD.pdf. All other relevant data are included in the paper (accessed on 24 November 2022).

## Supplementary Materials

The following supporting information can be downloaded at: https://www.mdpi.com/article/10.3390/ijerph20010844/s1, Figure S1: Use of cardiac surgery procedures among people with diabetes by months for years 2019 and 2020; Table S1: International Classification of Disease, 10th edition, (ICD-10) codes for the clinical diagnoses and procedures used in this investigation; Table S2: Multivariable Logistic regression models to assess the change from 2019 to 2020 in the in-hospital mortality among people with diabetes according to surgical cardiac procedures; Table S3: Characteristics of patients with diabetes who underwent a coronary artery bypass graft (CABG), percutaneous coronary intervention (PCI), open surgical valve replacement (OSVR), and transcatheter valve implantation (TCVI) from 2017 to 2019 in Spain. Analysis of the Spanish National Hospital Discharge Database; Table S4: In hospital mortality of patients with diabetes who underwent a coronary artery bypass graft (CABG), percutaneous coronary intervention (PCI), open surgical valve replacement (OSVR), and transcatheter valve implantation (TCVI) from 2017 to 2019 in Spain. Analysis of the Spanish National Hospital Discharge Database; Table S5: Multivariable Logistic regression models to assess the change from 2017 to 2020 in the in-hospital mortality among people with diabetes according to surgical cardiac procedures
