# Supplementary material for: Use of Cardiac Procedures in People with Diabetes during the COVID Pandemic in Spain: Effects on the In-Hospital Mortality
Source: Int J Environ Res Public Health. 2023 Jan 2;20(1):844. doi: 10.3390/ijerph20010844 (PMC9819421; doi:10.3390/ijerph20010844)
Supplement: Supplementary file 1 [file ijerph-20-00844-s001.zip › ijerph-2087714-supplementary.pdf]

Figure S1. Use of cardiac procedures among people with diabetes by months for years 2019 and 2020.

A. Coronary artery bypass graft (CABG)

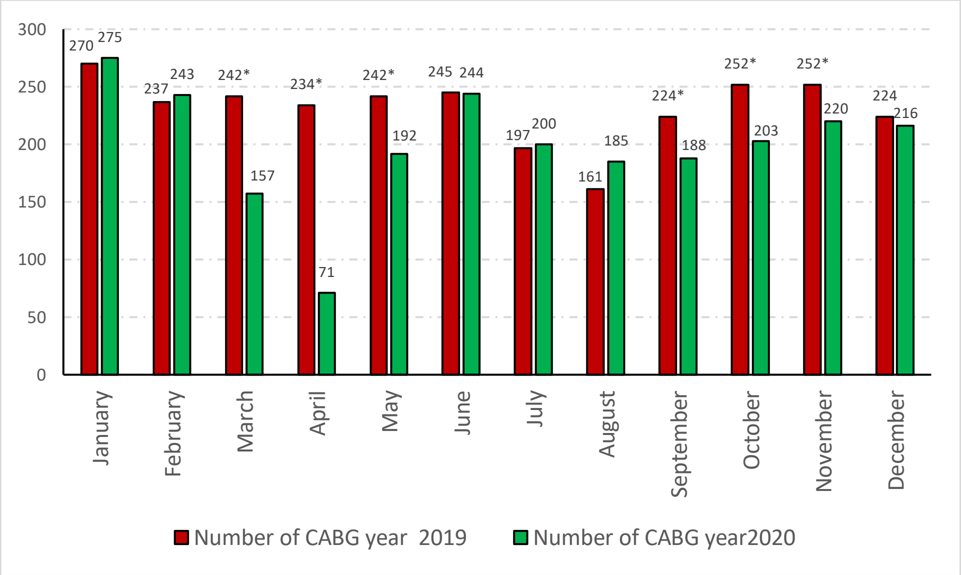

* Significant change that month

B. Percutaneous coronary intervention (PCI)

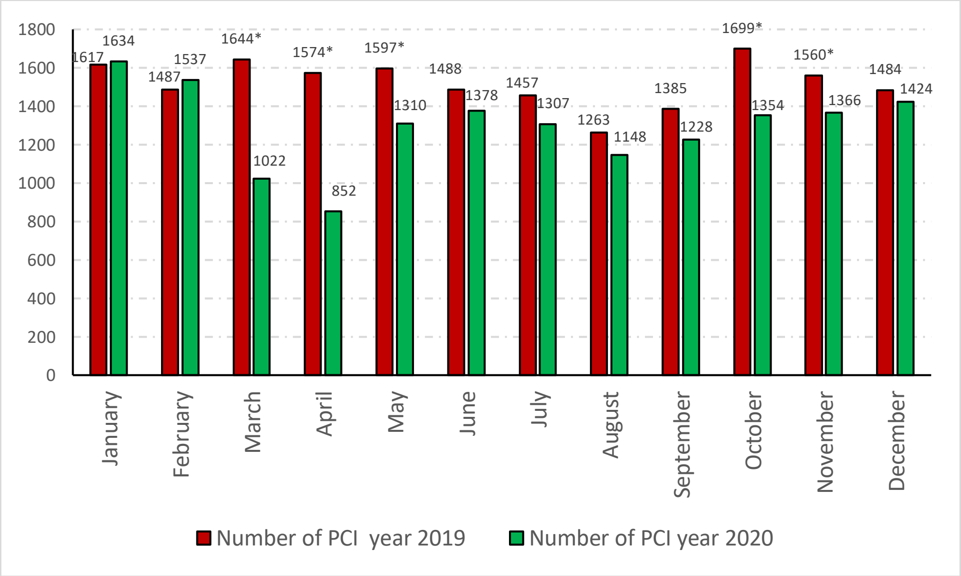

* Significant change that month

C. Open surgical aortic or mitral or tricuspid or pulmonary valve replacement (OSVR)

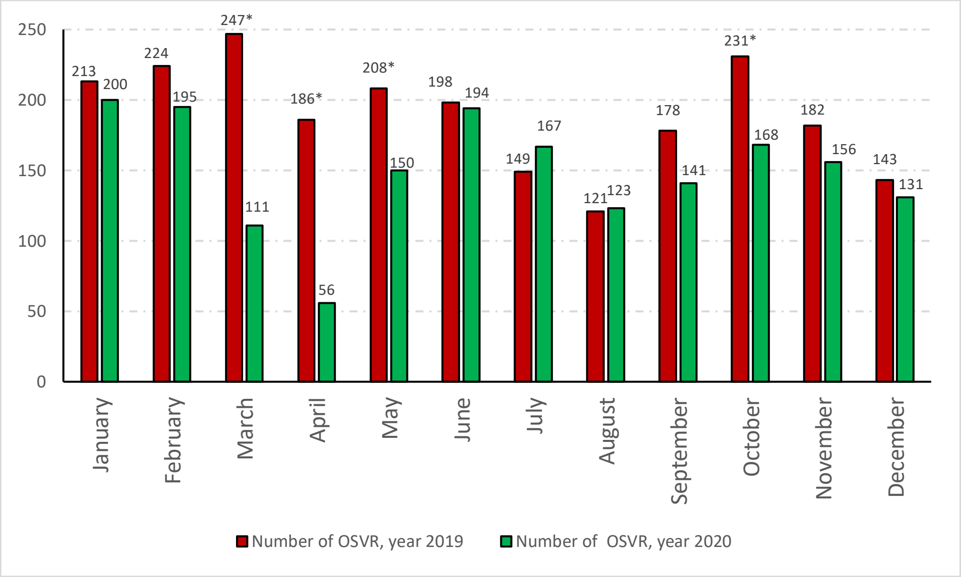

* Significant change that month

D. Trans-catheter aortic or mitral or tricuspid or pulmonary valve implantation (TCVI)

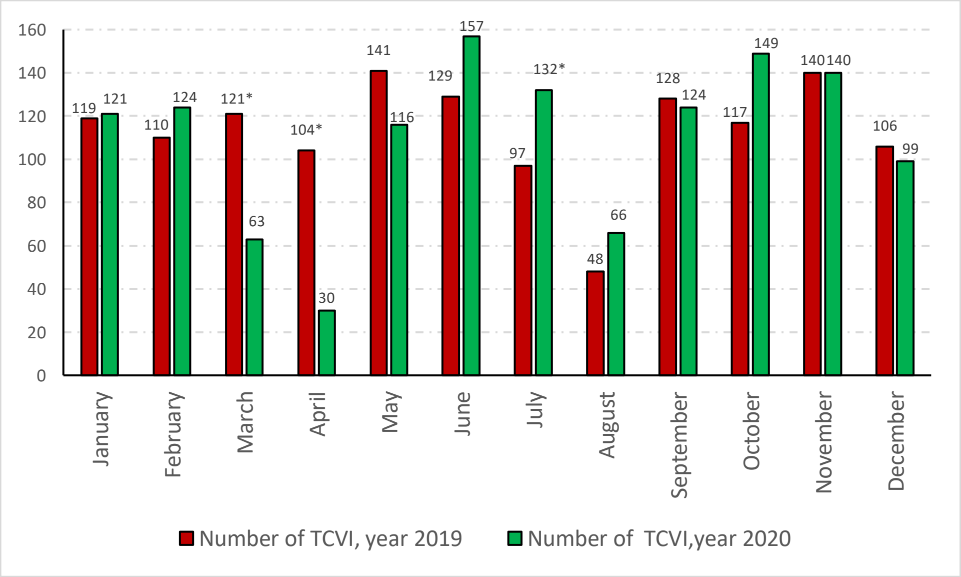

* Significant change that month

Table S1. International Classification of Disease, 10th edition, (ICD-10) codes for the clinical diagnoses and procedures used in this investigation.

**PROCEDURES**	**ICD-10 codes**
Percutaneous coronary intervention.	02703XX, 02704xx, 02713XX, 02714xx, 02723XX, 02724xx, 02733XX, 02734xx
Coronary artery bypass graft	02100XX, 02110XX, 02120XX, 02130XX
Open heart valve replacement	02RF0XX, 02RG0XX, 02RH0XX, 02RJ0XX
Trans-catheter valve implantation	02RF3xx; 02RG3xx; 02RH3xx; 02RJ3xx
Non-invasive mechanical ventilation	5A09357, 5A09457, 5A09557
Invasive mechanical ventilation	5A1945Z, 5A1955Z, 5A1935Z
Dialysis	5A1D xxx
**DIAGNOSIS**	**ICD-10 codes**
Diabetes	E10.xxx ,E11.xxx
Peripheral vascular disease	I70.x, I71.x, I73.1, I73.8, I73.9, I77.1, I79.0, I79.2, K55.1, K55.8, K55.9, Z95.8, Z95.9
Ischemic heart disease	I20-I25
Valvular heart disease	I05-I08, I33-I39
Atrial fibrillation	I48.0, I48.1, I48.2, I48.91
Heart failure	I09.9, I11.0, I13.0, I13.2, I25.5, I42.0, I42.5–I42.9, I43.x, I50.x, P29.0
Chronic kidney disease	N18
COVID 19	B34.2, B97.29, U07.1

Table S2. Multivariable Logistic regression models to assess the change from 2019 to 2020 in the in-hospital mortality among people with diabetes according to cardiac procedures

	Coronary artery bypass graft	Percutaneous coronary intervention.	Open Heart valve replacement	Trans-catheter valve implantation
Man	1	1	1	1
Women	1.94(1.41-2.66)	1.19(1.05-1.35)	1.79(1.38-2.32)	1.2(0.76-1.88)
Age (Continuous)	1.04 (1.01-1.07)	1.02 (1.00.1.04)	1.05 (1.02-1.08)	1.03 (1.00-1.07)
Charlson comorbidity index (Continuous)	1.45(1.35-1.55)	1.32(1.28-1.36)	1.36(1.27-1.47)	1.26(1.12-1.42)
2019	1	1	1	1
2020	1.01(0.76-1.35)	1.07(0.95-1.2)	1.06(0.82-1.37)	1.02(0.65-1.58)

Table S3. Characteristics of patients with diabetes who underwent a coronary artery bypass graft (CABG), percutaneous coronary intervention (PCI), open surgical valve replacement (OSVR), and transcatheter valve implantation (TCVI) from 2017 to 2019 in Spain. Analysis of the Spanish National Hospital Discharge Database.

		2017	2018	2019	p for trend
**CABG**	N	2789	2773	2780	0.325
	Men n (%)	2198(78.81)	2205(79.52)	2246(80.79)	0.185
	Women n (%)	591(21.19)	568(20.48)	534(19.21)	
	Age. mean (SD)	68.12(8.8)	68.02(8.82)	67.74(8.52)	0.226
	CCI index. mean (SD)	2.34(1.39)	2.44(1.5)	2.49(1.51)	<0.081
**PCI**	N	16437	17422	18255	<0.001
	Men n (%)	12050(73.31)	12934(74.24)	13421(73.52)	0.335
	Women n (%)	4387(26.69)	4488(25.76)	4834(26.48)	
	Age. mean (SD)	68.77(10.96)	68.68(10.97)	69.26(10.9)	<0.001
	CCI index. mean (SD)	2.61(1.43)	2.69(1.54)	2.75(1.57)	<0.011
**OSVR**	N	2387	2312	2280	0.103
	Men n (%)	1444(60.49)	1384(59.86)	1421(62.32)	0.067
	Women n (%)	943(39.51)	928(40.14)	859(37.68)	
	Age. mean (SD)	71.36(8.11)	70.94(7.99)	70.44(7.91)	0.159
	CCI index. mean (SD)	2.18(1.45)	2.19 (1.49)	2.21(1.43)	<0.051
**TCVI**	N	791	1100	1360	<0.001
	Men n (%)	410(51.83)	574(52.18)	716(52.65)	0.732
	Women n (%)	381(48.17)	526(47.82)	644(47.35)	
	Age. mean (SD)	79.73(6.61)	79.61(6.86)	79.64(6.78)	0.827
	CCI index. mean (SD)	2.64(1.57)	2.62(1.61)	2.62(1.61)	0.091

Table S4. In hospital mortality of patients with diabetes who underwent a coronary artery bypass graft (CABG), percutaneous coronary intervention (PCI), open surgical valve replacement (OSVR), and transcatheter valve implantation (TCVI) from 2017 to 2019 in Spain. Analysis of the Spanish National Hospital Discharge Database.

		2017	2018	2019	2020	p for trend
CABG	Both sexes, n (%)	109(3.91)	115(4.15)	106(3.81)	99(4.14)	0.892
	Men n (%)	83(3.78)	78(3.54)	76(3.38)	69(3.55)	0.885
	Women n (%)	26(4.4)	37(6.51)	30(5.62)	30(6.7)	0.875
	p for sex-differences	0.569	0.001	0.015	0.003	-
PCI	Both sexes, n (%)	518(3.15)	572(3.28)	639(3.5)	599(3.85)	<0.001
	Men n (%)	350(2.9)	387(2.99)	434(3.23)	411(3.56)	<0.001
	Women n (%)	168(3.83)	185(4.12)	205(4.24)	188(4.67)	0.027
	p for sex-differences	0.003	<0.001	0.001	0.002	-
OSVR	Both sexes, n (%)	160(6.7)	157(6.80)	140(6.14)	121(6.75)	0.007
	Men n (%)	91(6.3)	75 (5.42)	67(4.71)	69(6.07)	0.024
	Women n (%)	69(7.32)	71 (7.66)	73(8.5)	52(7.94)	0.234
	p for sex-differences	0.587	0.02	<0.001	0.129	
TCVI	Both sexes, n (%)	23(2.91)	36(3.27)	40(2.94)	42(3.18)	0.697
	Men n (%)	11(2.68)	15(2.85)	18(2.51)	17(2.67)	0.728
	Women n (%)	12(3.15)	21(3.66)	22(3.42)	25(3.65)	0.839
	p for sex-differences	0.696	0.450	0.325	0.312	

Table S5. Multivariable Logistic regression models to assess the change from 2017 to 2020 in the in-hospital mortality among people with diabetes according to cardiac procedures

	Coronary artery bypass graft	Percutaneous coronary intervention.	Open Heart valve replacement	Trans-catheter valve implantation
Man	1	1	1	1
Women	1.76(1.45-2.14)	1.2(1.1-1.3)	1.52(1.31-1.76)	1.11(0.8-1.54)
Age (Continuous)	1.04(1.02-1.05)	1.04(1.03-1.04)	1.02(1.01-1.03)	1.03(1.00-1.06)
Charlson comorbidity index (Continuous)	1.44(1.37-1.51)	1.35(1.32-1.38)	1.41(1.35-1.47)	1.29(1.18-1.4)
2017	1	1	1	1
2016	0.85(0.64-1.12)	1.05(0.93-1.19)	0.71(0.56-0.89)	0.76(0.44-1.31)
2019	0.87(0.64-1.16)	1.08(0.91-1.23)	0.74(0.58-0.94)	0.76(0.44-1.32)
2020	1.26(0.95-1.86)	1.12(0.97-1.36)	1.22(0.99-1.56)	1.11(0.8-1.54)
